# Editorial: Beneficial effects of fungal endophytes in major agricultural crops

**DOI:** 10.3389/fpls.2022.1061112

**Published:** 2022-11-14

**Authors:** Jorge Poveda, Paula Baptista, Soledad Sacristán, Pablo Velasco

**Affiliations:** ^1^ Institute for Multidisciplinary Research in Applied Biology (IMAB), Universidad Pública de Navarra, Arrosadía, Pamplona, Spain; ^2^ Centro de Investigação de Montanha (CIMO), Instituto Politécnico de Bragança, de Santa Apolónia, Bragança, Portugal; ^3^ Laboratório Associado para a Sustentabilidade e Tecnologia em Regiões de Montanha (SusTEC), Instituto Politécnico de Bragança, de Santa Apolónia, Bragança, Portugal; ^4^ Centro de Biotecnología y Genómica de Plantas (CBGP), Universidad Politécnica de Madrid (UPM), Instituto Nacional de Investigación y Tecnología Agraria y Alimentaria/Spanish National Research Council (INIA/CSIC), and Departamento de Biotecnología-Biología Vegetal, Escuela Técnica Superior de Ingeniería Agronómica, Alimentaria y de Biosistemas, Universidad Politécnica de Madrid (UPM), Madrid, Spain; ^5^ Group of Genetics, Breeding and Biochemistry of Brassicas, Misión Biológica de Galicia (MBG), Spanish National Research Council (CSIC), Pontevedra, Spain

**Keywords:** bioinoculants, plant growth promotion, abiotic stress tolerance, biological control agents, Brassicaceae

## Endophytic fungi and agriculture

Endophytic microorganisms are those that can dwell within plant tissues without any external sign of infection or other harmful effects on the host plants (Burragoni and Jeon, 2021). In recent decades, the important role that both bacterial and fungal endophytes play in plant growth and development, as well as in their ability to survive in their environment, has been identified (Burragoni and Jeon, 2021). Endophytic fungi can be found colonizing any plant organ, presenting a very different distribution and diversity among plants of different species, among plants of the same species, and even among organs of the same plant (Aamir et al., 2020). In crops, endophytic fungi act through different beneficial pathways, as biofertilizers promoting plant growth, as biological control agents of pathogens and pests or as inducers of tolerance under abiotic stresses, having great importance in the development of new strategies for sustainable agriculture (Aamir et al., 2020). These benefits for crops have been studied in the papers published in this Research Topic: promotion of plant growth in tomato (Paradza et al.), cotton (Silva et al.) and wheat (Asim et al.), increased tolerance under salt stress in tritordeum and perennial ryegrass (Toghueo et al.), as biological control agents against pathogenic fungi through antibiosis and mycoparasitism (Silva et al.), or as insecticidal agents through activation of systemic plant defenses (Paradza et al.; Agbessenou et al.), among others.

Despite their benefits, the use of endophytic fungi in agriculture is still very low, compared to the great number of fungi described with beneficial activity for crops. This large gap is a consequence of different factors, such as the strict regulatory standards for the registration and commercialization of new agricultural bioinoculants, the lack of studies showing the efficacy of the endophytes under field conditions, or the difficulty in mass production of inoculums (Murphy et al., 2018; Chitnis et al.). However, the number of patents with endophytic fungi is increasing exponentially in recent years, mainly with respect to their agricultural, industrial, environmental and medical use (Al-Ani, 2019). For their use as secondary metabolite producers and biotransforming agents, from January 2001 to December 2019, 245 patents were worldwide registered (Torres-Mendoza et al., 2020), while for their agricultural use, from January 1988 to December 2019, 185 patents were worldwide registered (Ortega et al., 2020).

## Endophytic fungi as plant growth promoters in crops

One of the main benefits obtained by crops with the use of endophytic fungi is the promotion of plant growth, resulting in higher agricultural productivity. The mechanisms of plant growth promotion developed by endophytic fungi include the increasing access to nutrients (nitrogen, phosphorus, potassium, zinc, iron, etc.), production of plant hormones, increase in water acquisition rate, and the modification of plant hormone levels (Poveda et al., 2021; Baron and Rigobelo, 2022).

When starting a plant growth promotion study using endophytic fungi, it is important to determine whether the isolates used are able to colonize new plants when reinoculated. In the work carried out by Paradza et al., using 16 isolates of endophytic fungi and applying them to tomato (*Solanum lycopersicum*) and pea (*Pisum sativum*) seedlings, only nine isolates colonized tomato and seven isolates colonized pea, and only four isolates (of the genera *Hypocrea* and *Trichoderma*) were able to colonize the tissues of both plants. Moreover, of these four isolates, only *T. atroviride* is able to promote plant growth, and only on tomato seedlings. Among the mechanisms possibly involved in the ability to promote plant growth by *Trichoderma*, the work of Silva et al. identifies phosphate solubilization as the main responsible. Under greenhouse conditions, these authors succeeded in promoting cotton (*Gossypium hirsutum*) plant growth by *T. asperelloides* and *T. lentiforme* isolates.

Endophytic fungi can also modify plant growth through direct action on their hormonal pathways. Using wheat (*Triticum aestivum*) plants and the endophytic fungus *Fusarium oxysporum*, Asim et al. reported plant growth promotion based on increased aerial and root biomass, increased root and stem length, increased index vigor, and increased germination. This plant growth promotion was associated with increased plant levels of the hormones auxins, gibberellins and salicylic acid, in addition to a decrease in the hormones abscisic acid and jasmonic acid. However, the same isolate may be involved in the growth inhibition of other plants. Indeed, the isolate *F. oxysporum* inhibited the wild weed *Avena fatua* (competitor with the wheat crop) in all growth parameters analyzed, causing increased oxidative stress in the plant and accumulation of flavonoids. This is due to the production of phytotoxic compounds with no effect on wheat plants, such as isovitexin, calycosin, quercetagetin, and dihydroxy-dimethoxyisoflavone (Asim et al.).

## Endophytic fungi as inducers of tolerance in crops under abiotic stresses

Crops face throughout their cycle different abiotic stresses, such as drought, salinity, extreme temperatures, heavy metal toxicity or nutritional deficiencies (Lata et al., 2018). These stresses significantly reduce plant growth and crop productivity, by causing ion toxicity, inhibition of photosynthesis, loss of membrane integrity or increasing generation of reactive oxygen species (Lata et al., 2018). Endophytic fungi can reduce the detrimental effect of these environmental conditions by inducing stress-tolerant plant responses or by direct synthesis of antistress biochemicals (Lata et al., 2018). In this sense, endophytic fungi can produce or induce plant production of antioxidant enzymes (catalase, superoxide dismutase, lipoxygenase, etc.), antioxidant secondary metabolites (phenols, flavonoids, etc.), organic solutes (proline, aspartic acid, etc.), and hormones (abscisic acid, gibberellins, etc.) (Tyagi et al., 2022).

A strategy used in recent years to obtain endophytic fungi with the ability to favor plant tolerance under abiotic stresses is the *in situ* isolation of these fungi in places with strong selective pressure derived from stress. Based on this hypothesis, Toghueo et al. obtained an isolate of *Diaporthe* from *Festuca rubra* subsp. *pruinosa*, a perennial grass adapted to rocky sea cliffs, where soil and nutrients are very limited, and exposure to salinity is continuous. Inoculation of tritordeum (*Triticum × Hordeum*) and perennial ryegrass with *Diaporthe* in the absence of abiotic stress promoted plant growth (higher leaf and root biomass), increased plant nutrient content (N, Ca, Mg and Fe) and increased auxin production. In addition, when plants were subjected to salinity stress (200 mM NaCl), the endophytic fungus reduced the detrimental effect of the stress by promoting plant synthesis and accumulation of proline, gibberellins and auxins, together with an increase in nutrient uptake by roots.

## Endophytic fungi as biological control agents against crop pathogens and pests

Biotic stresses (inflicted by pathogens, animals and weeds) cause annual crop losses by 20-40% worldwide, with specific losses caused by pathogens being 10-15% (Mohammad-Razdari et al., 2022). Endophytic fungi can reduce losses caused by pathogens in crops through different mechanisms of action. Indirectly, endophytic fungi can induce the defenses of its host plant, both local and systemically, against crop pathogens (Poveda et al., 2020a). Directly, endophytic fungi can act through the production of volatile and non-volatile antimicrobial compounds (antibiosis), competition for rhizospheric, phyllospheric and internal plant space and nutrients, and/or through direct attack on pathogens (mycoparasitism and nemato-parasitism) (Latz et al., 2018; Poveda and Baptista, 2021). One of the main endophytic fungi used as a biological control agent against crop pathogens are species from the genus *Trichoderma*. The work carried out by Silva et al. describes how different isolates of *Trichoderma* (*T. asperelloides* and *T. lentiforme*) are capable of acting as effective biological control agents against the cotton pathogen *Sclerorinia sclerotiorum*. In this work, both isolates were able to inhibit the growth of the pathogen mycelium by releasing volatiles, in addition to mycoparasitizing the sclerotia formed by *S. sclerotiorum*.

Endophytic fungi can also act through different mechanisms against insect pests. Directly, these fungi can infect insect tissues, or produce insecticidal compounds; and indirectly, can activate the host plant defenses and/or induce the production of compounds in the host plant with insecticidal or antifeedant activity (Bamisile et al., 2018; Poveda, 2021). The work carried out by Paradza et al., describes how the root inoculation of tomato and bean (*Phaseolus vulgaris*) plants with different isolates of the genus *Hypocrea* and *Trichoderma* causes a systemic increase in the defenses of the host plant. This plant defensive response causes greenhouse whiteflies (*Trialeurodes vaporariorum*) to reduce oviposition and adult longevity. Using the endophytic fungus *Trichoderma asperellum*, Agbessenou et al. managed to identify the mechanism involved in the reduction of the damage caused by the tomato leafminer (*Tuta absoluta*) in tomato plants. The presence of *T. asperellum* in the root tissues induced the systemic activation of a defensive response mediated by jasmonic acid, inducing the synthesis and release of methyl salicylate, a volatile substance that acts as a repellent against the herbivory of *T. absoluta*.

## The special case of the interaction between endophytic fungi and crops of the Brassicacea family

The Brassicaceae family includes plants of great scientific interest (such as *Arabidopsis thaliana*) and agronomic interest (such as those of the *Brassica* genus). This group of plants is characterized by the synthesis and accumulation in their tissues of a group of secondary sulfur metabolites, called glucosinolates (GSLs), which have a high antifungal capacity (specially when hydrolyzed to isothiocyanates) (Poveda et al., 2020b).

This family of plants is unable to form symbiotic relationships with mycorrhizal fungi. However, there are root endophytic fungi capable of supplying mycorrhizal functions (Hiruma et al., 2018). The review carried out by Poveda et al. stands out that the presence of GSLs in Brassicaceae plants could be one of the reasons for the absence of symbiotic relationships with mycorrhizal fungi. In this case, the endophytic fungi must tolerate or degrade the glucosinolates of the tissues of these plants in order to colonize them, providing important benefits to this group of crops. Precisely, the work carried out by Plaszkó et al. shows how the presence of metabolites, such as glucosinolates, is correlated with the diversity of endophytic fungi isolated from horseradish plants (*Armoracia rusticana*).

## Information gaps and future perspectives

Despite the benefits reported in agricultural crops by the use of endophytic fungi (plant growth promotion, tolerance to abiotic stresses and resistance to biotic stresses), there are still important aspects to be developed for these bioinoculants to become a reality in the market. Firstly, more studies should be carried out on the efficacy of these fungi in the field, since the situation of a controlled study in a greenhouse or growth chamber cannot be extrapolated to real agrosystem conditions. In addition, a biotechnology industry capable of mass-producing the fungal inoculum needed for the formulation of bioinoculants must be developed. All this must be accompanied by the establishment of clear national and international regulations that allow the use of endophytic fungi in agriculture in a safe and effective manner.

Endophytic fungi have been described in numerous studies as potential plant growth promoters by various mechanisms. In this sense, the vast majority of studies have been carried out by *in vitro* biochemical tests or *in planta* studies in greenhouses, so it is absolutely necessary to develop studies under field conditions, allowing us to really know the behavior of the fungus in the agrosystem and not its potential behavior. In addition, these studies would allow us to deepen our knowledge of why some endophytic fungi act as beneficial endophytes of certain plant species and as pathogens of others, an aspect of great interest to be investigated in the coming years.

The ability of endophytic fungi to increase plant tolerance under abiotic stresses is an aspect still little addressed and with great potential in agriculture, even more so in the current climate crisis. However, the role of these fungi as biological control agents of pathogens and agricultural pests has been widely developed in numerous works. In spite of this, it is required the development of field studies that really demonstrate the effectiveness of these microbial agents, in addition to the development of specific regulations that allow the safe and effective release of these microorganisms to the agrosystem.

A very interesting and successful aspect that is being developed in recent years regarding the use of endophytic fungi in agriculture is the development of co-inoculants, the study of synergies or antagonistic behaviors between the endophytic fungi used, and even how the endophytic fungus acts synergistically or antagonistically with the plant microbiota and vice versa. All these aspects still need a lot of research, but the use of new omics techniques will provide very important information in the coming years.

Finally, we would like to highlight the great potential of the study of endophytic fungi in Brassicaceae plants and specifically in *Brassica* crops, which have been very little studied so far. In addition to the description of new fungal species with great agricultural potential, Brassicaceae plants represent a unique model in the study of fungal-plant interactions, both in the understanding of why these plants are colonized by endophytic fungi but not mycorrhized, and to understand why there are endophytic fungi that behave as beneficial or pathogenic depending on their host plant.

## Author contributions

JP wrote the first version of the manuscript. PB, SS and PV contributed to the manuscript correction and critical reading. All authors have read and agreed to the published version of the manuscript.

## Funding

JP thanks Grants for the Recualification of the Spanish University System for 2021-2023, Public University of Navarra; Recualification Modality; Funded by the European Union –NextGenerationEU. PB is grateful to the Foundation for Science and Technology (FCT, Portugal) for financial support through national funds FCT/MCTES (PIDDAC) to CIMO (UIDB/00690/2020 and UIDP/00690/2020) and SusTEC (LA/P/0007/2020). SS is grateful to Plan Estatal de Investigación Científica, Técnica y de Innovación 2021-2023, Ministerio de Ciencia e Innovación of Spain, with grant number PID2021-123697OB-I00. PV is grateful to Xunta de Galicia (Spain) for financial support by project IN607A 2021/03.

JP and PB are grateful to the European Union’s Framework Programme for Research and Innovation, within the project PRIMA/0002/2018 (INTOMED - Innovative tools to combat crop pests in the Mediterranean) (Horizon 2020).

## Conflict of interest

The authors declare that the research was conducted in the absence of any commercial or financial relationships that could be construed as a potential conflict of interest.

## Publisher’s note

All claims expressed in this article are solely those of the authors and do not necessarily represent those of their affiliated organizations, or those of the publisher, the editors and the reviewers. Any product that may be evaluated in this article, or claim that may be made by its manufacturer, is not guaranteed or endorsed by the publisher.
